# Recurrent Bullous Pemphigoid: A Case Report

**DOI:** 10.7759/cureus.113618

**Published:** 2026-07-29

**Authors:** Ramachandra Reddy Gowda Venkatesha, Karthik Rajaram Mohan, Mirnalini Aravind, Vasu Sridharrao, Nandhini Chandran

**Affiliations:** 1 Oral Medicine and Radiology, Vinayaka Mission's Sankarachariyar Dental College, Vinayaka Mission's Research Foundation (Deemed to be University), Salem, IND

**Keywords:** bullous pemphigoid (bp), dapsone treatment, diabetes type 2, tense bullae, topical triamcinolone acetonide

## Abstract

Recurrent bullous pemphigoid is a rare autoimmune vesiculobullous disorder caused by bullous lesions on the body and areas of ruptured bullae that leave raw erythematous regions, resulting in a burning sensation in the oral cavity. The article enlightens the occurrence of bullous pemphigoid in a 68-year-old male who complained of a burning sensation in his oral cavity. The diagnosis was exclusively based on history and clinical examination without any intervention by histopathological or immunofluorescent tests.

## Introduction

Bullous pemphigoid has an annual incidence of six to 13 instances per 1,000,000 individuals in the population among the United States and 12 to 13 per 1,000,000 in the population of Central Europe [1]. The autoantibodies target two specific components of the basal keratinocyte hemidesmosomal proteins, namely BP antigen 230 (BPAG1) and BP antigen 180 (BPAG2 or type XVII collagen) [1,2]. These antigens are crucial components of the adhesion complexes that facilitate the binding of epithelial and stromal cells. Upon binding to their target antigen, autoantibodies initiate an inflammatory response by activating mast cells and complement leads to the production of inflammatory cells by eosinophils and neutrophils, resulting in the release of proteolytic enzymes that cause damage to the epidermal-dermal interface [1,2,3].

In 1953, Walter Lever distinguished bullous pemphigoid (BP) from pemphigus. Histopathological features of pemphigus, such as acantholysis, cell adherence loss between keratinocytes, and intraepidermal split development, were detailed by him. When he noticed that a sub-epidermal split was common, he came up with the term "pemphigoid" to describe the illness [4].

Antibodies that target BP180, a type XVII collagen, hemidesmosomes HD1 (BPAG1), a 230-kDa glycoprotein on chromosome 6p11-6p12, and HD4 (BPAG2), a 180-kDa hemidesmosome glycoprotein on chromosome 10q 24.3, produce the chronic autoimmune illness known as bullous pemphigoid (BP). A subepithelial blister forms because of the 230 kDa BP antigen, a non-collagenous protein of the Plakin family found in basal keratinocytes and an autoantigen in bullous pemphigoid (BP). This antigen is found in the intracellular hemidesmosome plaque. The attachment of basal keratinocytes to the underlying basement membrane is stabilized when the 180-kDa BP antigen, a type XVII collagen transmembrane protein, binds with α6Η4 integrin and extends from the intracellular compartment of basal cells to the extracellular space [4].

"Pemphix" means "pustule" in Greek, and "oid" means "to resemble"; hence, the term "pemphigoid" is formed. Once pruritic papules appear on the extremities, BP lesions will appear in the mouth. It is more difficult for the dentist to diagnose these lesions. When the immune system destroys the hemidesmosome proteins anti-BP 180 and BP 230, they target the basement membrane zone and cause subepithelial blisters to form, leading to chronicity and numerous ulcers. Oral lesions do not appear until the itchy skin rashes have progressed. The incidence of bullous pemphigoid among the Indian population is 0.75%, and the mean age of occurrence is 62.5 +/-2.2 years, and associated comorbidities include type II diabetes and hypertension [4]. An additional risk factor for developing bullous pemphigoid (BP) is a history of psoriasis or lichen planus. An oral physician should not ignore the presence of bullous pemphigoid (BP) and should instead seek a comprehensive medication history and clinical assessment [4].

There are multiple independent but interdependent phases in the etiopathogenesis of bullous pemphigoid. Among these were the following phases. Acceleration of complement metabolism. When antibodies directed against hemidesmosomal glycoprotein antigens, such as BP180 and BP230, bind, complement activation takes place. Neutrophils and eosinophils secrete protease enzymes. In bullous pemphigoid (BP), neutrophils and eosinophils secrete proteases that are attracted to inflammatory response sites independently of complement activation. A process wherein keratinocytes micropinocytose anti-BP180. Through micropinocytosis, anti-BP180 IgG may cause keratinocyte cell membrane BP180 internalization [4]. Apoptosis-inducing tumor necrosis factor TNF-related weak signal (TWEAK). The connection between TNF-related weak signal (TWEAK) and fibroblast growth factor-inducible 14 (Fn14) helps induce blister formation in bullous pemphigoid (BP). Tumor necrosis factor-related weak signal (TWEAK) is a member of the tumor necrosis factor (TNF) superfamily [4].

In bullous pemphigoid (BP), the immunological reactions are triggered by cytokines IL-5, -4, and -13, activated by eosinophilic cationic protein, the main basic protein found in eosinophils. Furthermore, research has shown that eosinophils are the primary producers of tissue factor, which promotes blood coagulability and can cause pulmonary embolism in susceptible individuals [4]. Activated white blood cells' intercellular adhesion molecule 1 (ICAM-1). The chemicals generated by lymphocytes inflaming the skin, IFN-γ and TNF-α, stimulate the production of ICAM-1 on keratinocytes in the basal and lower suprabasal layers. ICAM-1 and HLA-DR output by keratinocytes is stimulated by activated lymphocyte IFN-γ, promoting inflammatory and allergic epidermal responses [4]. Chymase is produced by mast cells. In the same secretory granules as tryptase, the mast cells tryptase/chymase (MCTC) subset of mast cells store chymase, a monomeric protease with a molecular weight of 30 kDa. In bullous pemphigoid (BP), chymase helps break the dermal-epidermal junction. The hemidesmosomal proteins BP180 and BP230 aid in connecting the basement membrane and underlying connective tissue; however, in BP, an autoimmune illness that manifests over time, autoantibodies are produced and attack these proteins. Antibodies bind to the basement membrane, creating the complement cascade that brings in eosinophils and neutrophils [4].

This paper details the experience of a 68-year-old male patient affected by bullous pemphigoid with associated comorbidities like obesity and hypercholesterolemia.

## Case presentation

A 68-year-old male reported to our Department of Oral Medicine and Radiology with a chief complaint of a burning sensation in his mouth for the past month. A general examination revealed his body weight was 97 kg. His medical history revealed that he was a known diabetic and did not take regular antidiabetic medication. Extraoral examination revealed pigmentation on the extensor aspect of his right forearm. An intact blister is seen on the skin over his abdomen (Figure 1A, 1B).

**Figure 1:**
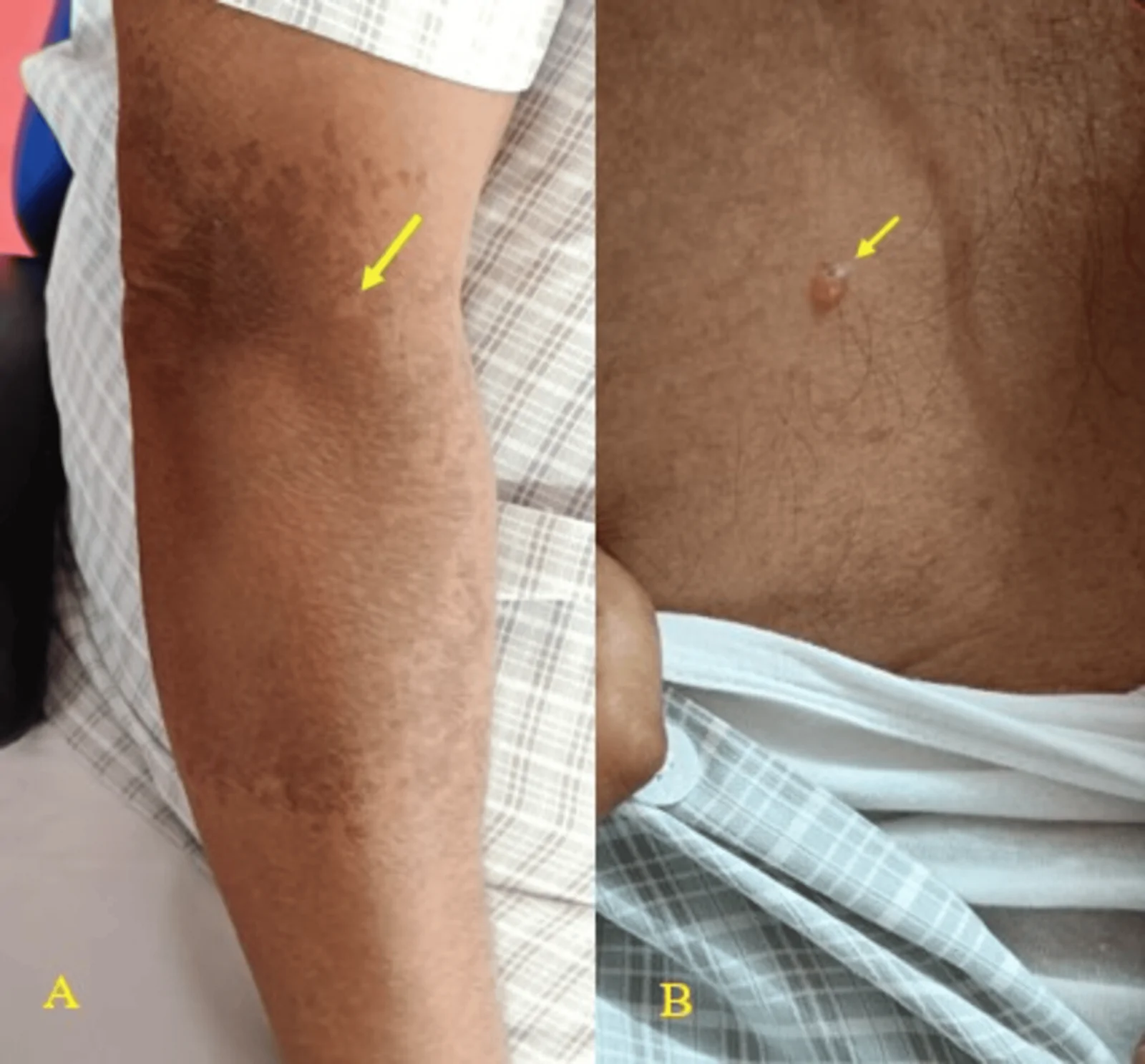
Clinical images A: An extraoral examination revealed discrete melanin pigmentation on the extensor aspect of the forearm (yellow arrow); B: Presence of a blister on the skin of the abdomen (yellow arrow). Patient consent was obtained for publication of his clinical images.

A ruptured vesicle was also noted along the lower border of the vermilion border of the lip near the cutaneous and vermilion border of the lip junction. Intraoral examination revealed bright-red areas of attached gingiva concerning his right maxillary central and lateral incisor region due to erosion caused by a recent rupture of vesicle on the gingiva (Figure 2A, 2B).

**Figure 2:**
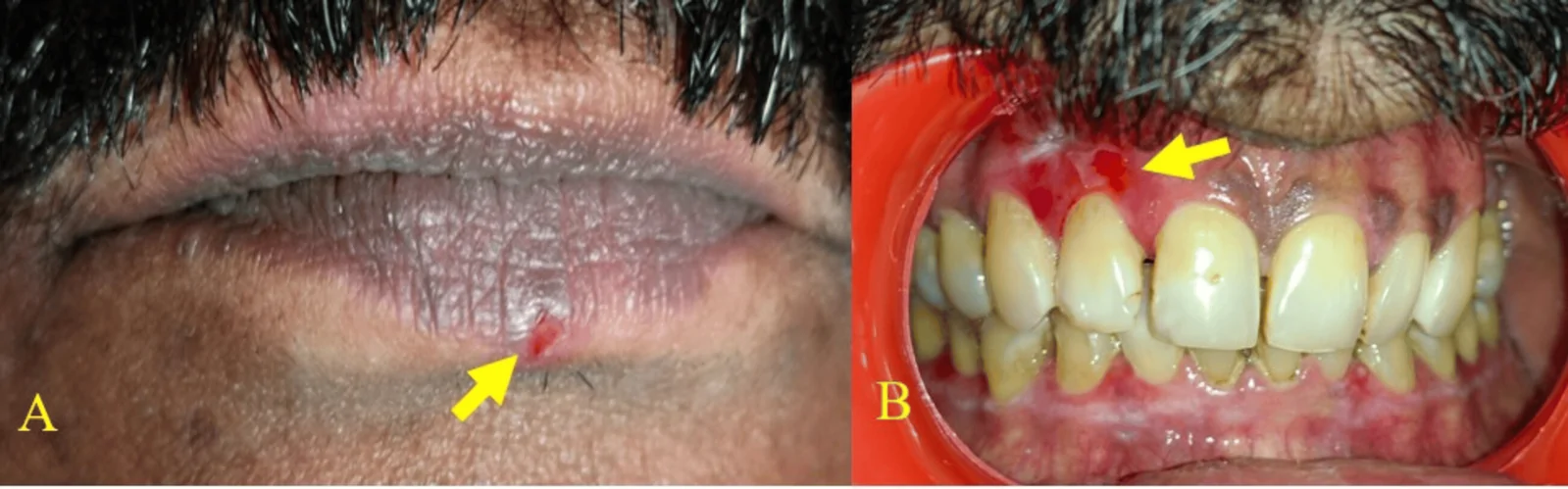
Clinical images A: Ruptured blister on the lower vermilion border of the lip near the dermal-lip junction (yellow arrow); B: A bright-red area of erosion on the labial aspect of attached gingiva concerning the right maxillary lateral and canine regions (yellow arrow).

His total cholesterol was 221 mg/dl; high-density lipoprotein (HDL) and low-density lipoprotein (LDL) cholesterol were 40 mg/dl and 110 mg/dl, respectively. His postprandial blood glucose level was 260 mg/dl, and glycated hemoglobin (Hb%) was 7.2%, which revealed poor control over his blood glucose levels.

Based on the clinical features, a provisional diagnosis of bullous pemphigoid was made. The differential diagnosis was desquamative gingivitis, lichen planus affecting the gingiva. There was no peeling of gingiva in our case. Hence, desquamative gingivitis was ruled out. The lichen planus is characterized by Wickham’s striations, which do not appear in bullous pemphigoid. Since the patient was diabetic, systemic steroids were contraindicated in our case. The patient was prescribed a tablet dapsone (100 mg for 14 days). Kenalog in orabase gel (triamcinolone acetonide), thrice daily for two weeks. The patient was followed up after two weeks; the lesion on the attached gingiva regressed. The patient reported no burning sensation in his oral cavity, but the bullae in the skin of his abdomen ruptured, darkened, and persisted (Figure 3A, 3B, 3C).

**Figure 3:**
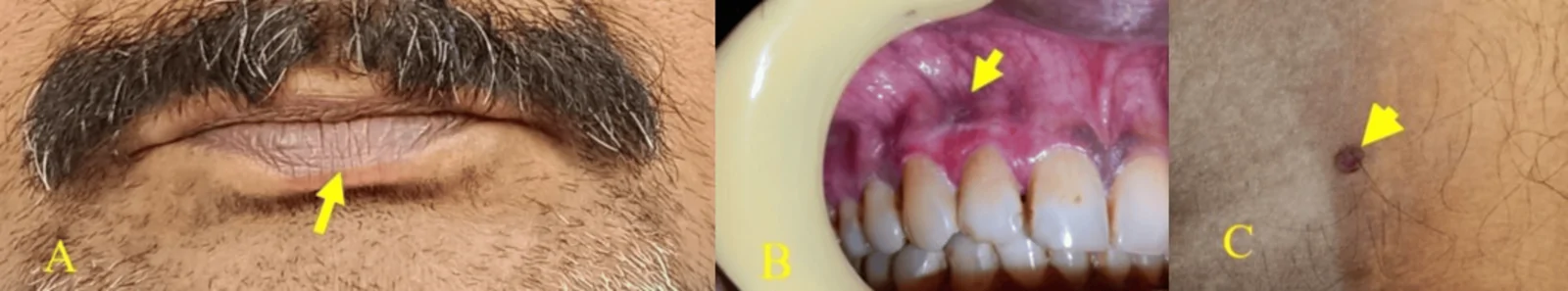
Follow-up clinical evaluation after two weeks A: Revealed healed blister near the dermal-vermilion border of the lip (yellow arrow); B: Healed areas of erosion in relation to the labial aspect of the attached gingiva concerning the right maxillary central and lateral incisor region (yellow arrow); C: Ruptured and darkened bullae on the skin of the abdomen (yellow arrow).

The patient was referred to a dermatologist for management of his persisting lesions on the skin. The patient was advised to do periodic follow-up. The patient was prescribed Caladryl® (Piramal Enterprises, Mumbai, India) containing calamine 8% w/w and diphenhydramine hydrochloride 1% w/w, 95% ethanol 2.37% w/w, for his pruritic skin blisters. The patient was relieved from pruritis. Tab roxuvostatin 5 mg once daily for a month for regulation of his high serum cholesterol levels.

Clinical significance

The paper highlights that associated comorbidities such as obesity and hypercholesterolemia are at risk for recurrent bullous pemphigoid.

## Discussion

Patients with bullous pemphigoid experience pruritus alone or associated with papular urticarial lesions in the prodromal phase [1]. Patients with bullous pemphigoid typically present with bullae occurring in the abdomen, back of the neck, forearms, trunk, and thigh regions. The bullae are initially filled with fluid, appear shiny, later rupture, and get darkened over a period of time [2]. Diagnosis of the bullous pemphigoid mainly depends on the clinical findings [1,2]. There is a possible connection between type 2 diabetes mellitus (T2DM) and a heightened likelihood of high blood pressure (BP). Drugs such as gliptins, terbinafine, and camrelizumab are known to induce bullous pemphigoid. The medications that can cause drug-induced bullous pemphigoid include diuretics such as bumetanide, furosemide, and spironolactone. Analgesics such as ibuprofen, aspirin, D-penicillamine, and antibiotics such as ciprofloxacin, amoxicillin, psycholeptics like phenothiazines, angiotensin converting enzyme (ACE) inhibitors such as captopril, antidiabetic agents like metformin, and dipeptidyl peptidase IV inhibitors (vildagliptin, saxagliptin, linagliptin, alogliptin, sildagliptin), and programmed death-1 (PD-1) inhibitors such as pembrolizumab, nivolumab, camrelizumab, sacubitril/valsartan, and repeated COVID-19 vaccinations [3-8]. Bullous pemphigoid is linked to the renal allograft rejection following a kidney transplant [9]. Bullous pemphigoid skin lesions can be easily misinterpreted due to perifollicular melanocyte regeneration in patients with darker Fitzpatrick type IV (Asian origin, medium-dark brown skin color, hardly burns, tans easily) skin tones [10]. Dipeptidyl-peptidase 4 inhibitors (DPP4i) are often prescribed medications for lowering blood sugar levels that have a strong connection to bullous pemphigoid (BP) with the involvement of the esophagus [11]. Dysphonia and dysphagia occur when a bullous pemphigoid affects the larynx [12]. Our patient was known to be diabetic and did not take regular antidiabetic medication. Whenever his blood glucose was poorly controlled, there was an exacerbation of bullous pemphigoid. Trastuzumab used as an adjuvant targeted therapy along with radiotherapy in the treatment of grade 3 invasive mammary carcinoma of the breast resulted in bullous pemphigoid, which was beneficial with dupilumab therapy [13]. Alsabbagh M, Bava A, and Ansari S (2023) reported a case of pembrolizumab-induced bullous pemphigoid [14].

Oral Janus kinase inhibitors such as baricitinib at a dose of 4 mg per day for twelve weeks and 15 mg of upadacitinib for three months proved substantial efficacy in the management of bullous pemphigoid [15]. Omalizumab is used in resistant cases of bullous pemphigoid [16]. Abrocitinib-A, a Janus kinase inhibitor, was beneficial to patients with refractory bullous pemphigoid [17]. Guselkumab is successful in refractory bullous pemhigoid [18]. The diacron-reactive oxygen metabolite levels determined by the diacron-reactive oxygen metabolite test reflect the disease severity in bullous pemphigoid [19]. Five sessions of double filtration plasmapheresis are beneficial in diabetic patients on chronic hemodialysis with severely relapsed bullous pemphigoid under biotherapy with omalimumab [20].

## Conclusions

A thorough knowledge of the clinical findings on recurrent bullous pemphigoid is essential. Dentists must understand the clinical features of this chronic autoimmune disease. Bullous pemphigoid is typically a chronic vesiculobullous disorder with remissions and exacerbations and an unpredictable prognosis. Skin precautions are crucial in managing bullous pemphigoid to prevent complications, promote healing, and reduce the risk of infection. Prompt referral to a dermatologist is essential in patients with bullous pemphigoid.
